# Predictors of Bone Mineral Density Improvement after Parathyroidectomy for Secondary Hyperparathyroidism: A Retrospective Single-Center Analysis

**DOI:** 10.1007/s00268-021-06186-1

**Published:** 2021-06-16

**Authors:** Manabu Okada, Yoshihiro Tominaga, Toshihide Tomosugi, Takahisa Hiramitsu, Toshihiro Ichimori, Tetsuhiko Sato

**Affiliations:** 1 Department of Transplantation and Endocrine Surgery, Nagoya Daini Red Cross Hospital, 2-9 Myoken-cho, Showa-ku, Nagoya, 4668650 Japan; 2 Department of Endocrinology, Nagoya Daini Red Cross Hospital, Nagoya, Japan

## Abstract

**Background:**

Parathyroidectomy (PTx) reportedly increases bone mineral density (BMD) in patients with severe secondary hyperparathyroidism (SHPT). To date, however, there has not been sufficient evidence on predictors of BMD improvement post-PTx for SHPT, an issue the present retrospective cohort study aimed to address.

**Methods:**

A total of 173 SHPT patients who underwent total PTx with forearm autograft between 2009 and 2017 were included in the present study. Demographic information, perioperative laboratory data and pre- and post-PTx BMD values (measured by dual-energy X-ray absorptiometry) were collected from their medical records. The change in BMD post-PTx in the lumbar spine was evaluated as the primary outcome. Then, a multivariate logistic regression analysis was performed for a ≥ 10% increase in BMD post-PTx.

**Results:**

Overall, the median BMD in the lumbar spine was increased by 8.7% post-PTx. The multivariate logistic regression analysis revealed that age ≥ 70 years (*P* = 0.005; odds ratio [OR], 0.138; 95% confidence interval [CI]: 0.034–0.555), serum Ca level (*P* = 0.017; OR, 0.598; 95% CI: 0.392–0.911) and pre-PTx BMD in the lumbar spine (*P* = 0.003; OR, 0.013; 95% CI: 0.001–0.229) were negatively associated with a ≥ 10% increase in BMD post-PTx.

**Conclusion:**

Our study demonstrated that presurgical age, serum Ca levels and BMD values could better predict an improvement in BMD post-PTx in SHPT patients.

**Supplementary Information:**

The online version contains supplementary material available at 10.1007/s00268-021-06186-1.

## Introduction

The risk of fracture in patients with chronic kidney disease (CKD) is significantly higher than that in the general population [1, 2]. Some of the known risk factors for fracture in CKD patients include older age, diabetes, female sex and secondary hyperparathyroidism (SHPT)—which is commonly found in CKD patients as a cause of renal osteodystrophy [3, 4]. The mechanisms whereby SHPT increases the risk of fracture mainly involve osteitis fibrosa and osteoporosis following persistently elevated parathyroid hormone (PTH) levels [5–8].

Parathyroidectomy (PTx) has been reported to reduce the risk of fracture and increase bone mineral density (BMD) in patients with severe SHPT and remains the most effective treatment option for advanced SHPT [9, 10]. However, although some SHPT patients experience a significant increase in BMD post-PTx, others do not achieve marked improvement in their BMD.

A few studies have reported some predictors of post-PTx BMD changes [11–13]. However, the validity of the multivariate analyses conducted in these studies is hampered by the relatively small sample sizes. To help identify potential predictors of PTx-induced improvement in BMD, we conducted the present retrospective study with a much larger population of SHPT patients who underwent total PTx with forearm autograft.

## Material and methods

### Study design and subjects

All patients who underwent PTx for SHPT at Nagoya Daini Red Cross Hospital (Nagoya, Japan) between January 2009 and December 2017 were retrospectively analyzed. Patients who had their initial PTx at other institutions, missing BMD data, persistent SHPT post-PTx (intact PTH levels ≥ 60 pg/mL on the first day post-PTx) and renal transplantation within 1-year post-PTx were excluded from the present study. For the patients who met the inclusion criteria, data on demographic and clinical characteristics, such as age, sex, dialysis vintage, renal diagnosis, perioperative laboratory findings, pre- and post-PTx BMD and pre- and post-PTx T-scores were collected from their electronic medical records. Preoperative blood sampling tests were performed at the first visit of the patients to the Department of Endocrine Surgery at Nagoya Daini Red Cross Hospital within 3 months before PTx. In the present study, the percent change in BMD of the lumbar spine was evaluated as the primary outcome. The improvement in BMD was defined as a postoperative BMD increase of ≥ 10%. To estimate predictive factors for BMD improvement induced by PTx, patients were divided into two groups according to post-PTx BMD change in the lumbar spine as follows: I-BMD group, comprising of patients with a ≥ 10% increase in BMD and NI-BMD group, comprising of patients with a < 10% increase in BMD. The abovementioned patient characteristics and laboratory data were compared between the two groups, followed by multivariate analyses. The present study was approved by the Institutional Review Board of Nagoya Daini Red Cross Hospital and was conducted according to the Declaration of Helsinki. Consent for participation could not be obtained from each patient, given the retrospective nature of the present study. Instead, the study protocol and supporting contents are available on the webpage of Nagoya Daini Red Cross Hospital. The opportunity and rights of the patients to drop out of the study were guaranteed.

### Surgical procedure and postoperative management

The routine operative technique was total PTx with forearm autograft, which involved resection of the thymic tongue followed by insertion of approximately 90 mg of the parathyroid tissue into the forearm muscles. Surgical indications for SHPT were established according to the clinical practice guidelines issued by the Japanese Society for Dialysis Therapy as follows [14]: high PTH levels (intact PTH, ≥ 500 pg/mL), hyperphosphatemia (serum phosphorus [sP], ≥ 6.0 mg/dL), or hypercalcemia (serum calcium [sCa], ≥ 10.0 mg/dL) when refractory to medical treatment and severe osteopenia, pathological fractures, renal stones, parathyroid gland diameter of > 10 mm as measured on imaging and severe SHPT-related symptoms. After PTx, calcium (Ca) and vitamin D supplementation with oral Ca carbonate 1.5–12 g/day and alfacalcidol 1–3 µg/day was initiated when the sCa level decreased to < 9 mg/dL. In cases with severe hypocalcemia or tetany, intravenous Ca was also administered. The doses of the Ca and vitamin D supplementation were adjusted to maintain the sCa levels within a range of 8.0–9.0 mg/dL. After discharge, Ca and vitamin D supplementation was continued at optimal doses at dialysis clinics. Evaluation using dual-energy X-ray absorptiometry (DEXA) was scheduled 1-year post-PTx, and low BMD (T-score, <  − 2.5) was considered diagnostic for osteoporosis.

### Measurements

BMD values were measured by DEXA (QDR Discovery-A; Hologic Inc., Tokyo, Japan). In the measurement of lumbar spine, the L2–L4 vertebrae were laterally scanned, with the mean of these three measurements determined as the lumbar spine BMD. Anteroposterior DEXA of the lumbar spine was not included in the assessment to avoid the influence of calcification of the descending aorta. Pre- and post-PTx BMD values were calculated within 6 months before PTx and 1-year post-PTx, respectively. T-scores represented the numbers of standard deviations from the mean young healthy adult’s BMD. Intact PTH levels were measured by an enzyme immunoassay (TOSOH Company, Tokyo, Japan). The sCa and sP levels were measured using the standard methods. When the serum albumin levels were < 4.0 mg/dL, the sCa levels were corrected as follows [15]:

Corrected Ca (mg/dL) = measured sCa (mg/dL) − albumin (g/dL) + 4.0

The percent change in BMD post-PTx was calculated as follows:

Percent change of BMD (%) = (post-PTx BMD [g/cm^2^] − pre-PTx BMD [g/cm^2^]) × 100/pre-PTx BMD (g/cm^2^).

### Statistical analysis

Nominal variables were analyzed using Pearson’s chi-squared test and the Mann–Whitney* U* test was applied to compare continuous and ordinal variables. All results were presented as median and interquartile range. The Spearman rank correlation coefficient was used to evaluate the correlation among the variables (Electronic Supplementary Material [ESM] 1). We conducted a multivariate stepwise logistic regression analysis for the ≥ 10% increase in BMD in the lumbar spine post-PTx. In addition, the crude and multivariable-adjusted odds ratios (ORs) for the ≥ 10% increase in BMD in the lumbar spine for the categories of pre-PTx sCa levels and BMD values were also examined using logistic regression analysis. Furthermore, a multivariate linear regression analysis of the ratio of post-PTx BMD to pre-PTx BMD (post-PTx BMD-to-pre-PTx BMD ratio) in the lumbar spine, including other explanatory variables, was also performed to assess the robustness of the results of the logistic regression analysis (ESM 2). Logarithmic conversion was applied to the values of PTH, bone-specific alkaline phosphatase (BAP) and post-PTx BMD-to-pre-PTx BMD ratio for normal distribution in the linear regression. The percent change in BMD was not adopted as the objective variable because the percent change can take a negative value, which cannot be converted to logarithms (Log). Age was treated as a categorical variable in 10-year increments. SPSS version 23.0 for Windows (IBM Japan Ltd., Tokyo, Japan) and EZR version 1.40 for Windows [16] were used for statistical analyses. Two-tailed *P* values < 0.05 were considered statistically significant.

### Theory and calculation

In the present study, the BMD change in the lumbar spine rather than the radius was chosen as the primary outcome because the radius is a cortical bone-rich tissue that is likely to have less BMD improvement after PTx than the lumbar spine [11–13].

The rationale for defining a 10% or greater increase in BMD in the lumbar spine as a significant improvement in bone mass is based on the following evidence: Lu et al. reported a 12.2% increase in BMD of the lumbar spine 1 year after PTx [12]. Sharma also pointed out that the BMD change in the lumbar spine was 12.3% approximately 2 years after PTx [17]. By contrast, a 6%–9% increase in lumbar BMD induced by bisphosphonate has been reported to reduce the vertebral fracture risk by over 40% in a few years [18, 19]. Taken together, we ended up with the notion that a 10% or greater BMD increase is meaningful because DEXA values are known to fluctuate by about several percent [20].

## Results and discussion

### Preoperative characteristics

**Fig. 1:**
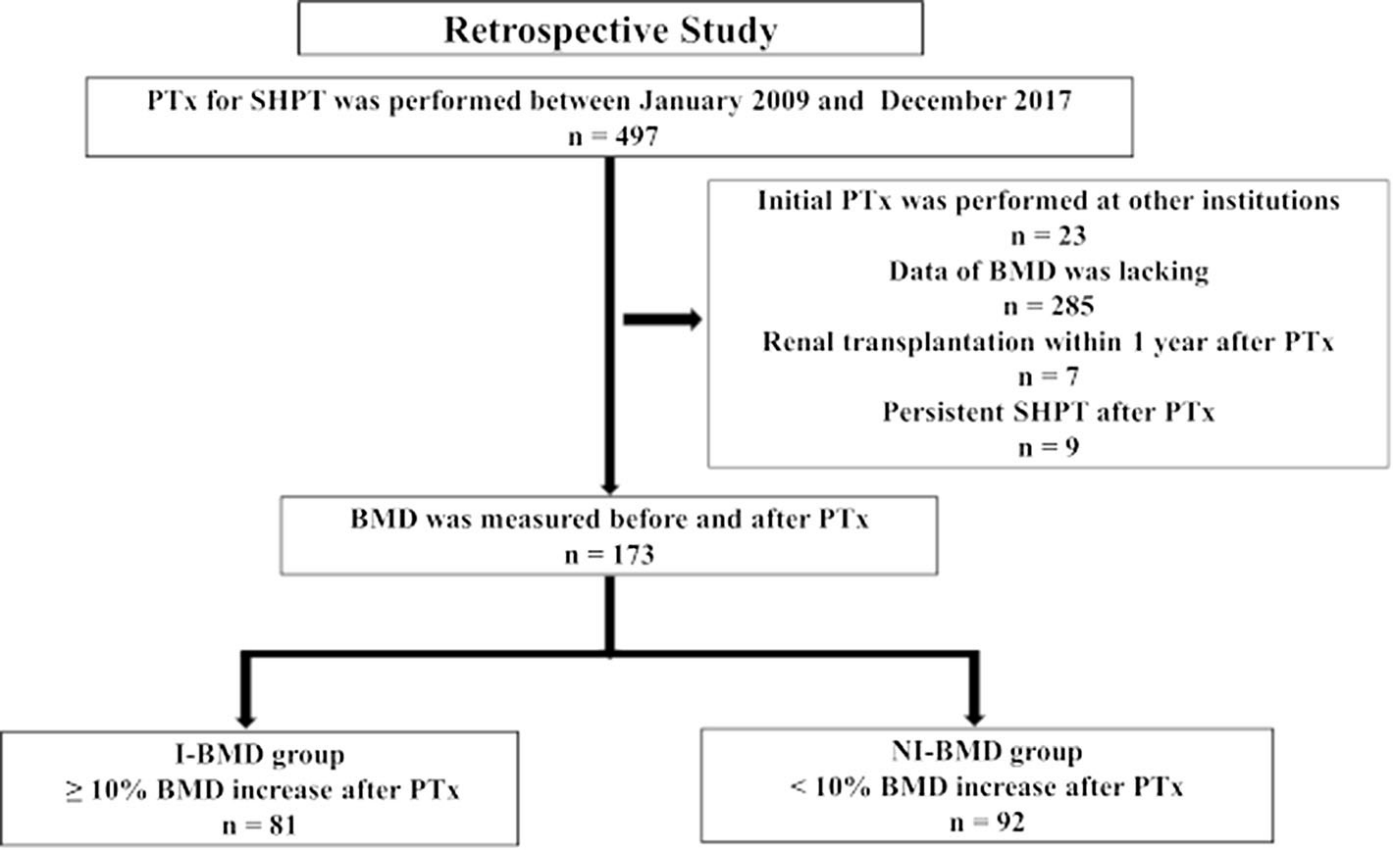
Study selection flowchart. *BMD*, bone mineral density; *I-BMD*, bone mineral density increased by 10% or more after parathyroidectomy; *NI-BMD*, bone mineral density increased by less than 10% after parathyroidectomy; *PTx*, parathyroidectomy

Figure 1 illustrates that 173 patients met the inclusion criteria and were included in the study. Of the 173 patients, 81 were assigned to the I-BMD group (comprising patients with a ≥ 10% increase in BMD) and 92 were assigned to the NI-BMD group (comprising patients with a < 10% increase in BMD). All the patients, except three were undergoing dialysis. Two patients (with forearm and jaw fractures, respectively) in the I-BMD group and three patients (with lumbar spine, hip and forearm fractures, respectively) in the NI-BMD group had a history of fracture prior to PTx. Significant differences in sCa level (9.9 vs. 10.4 mg/dL, *P* = 0.002), BAP (32.3 vs. 23.3 µg/L, *P* = 0.002), T-score at the lumbar spine and radius (− 3.0 vs. − 2.5, *P* = 0.047 and − 3.7 vs. − 3.2, *P* = 0.019, respectively) and the proportion of the patients treated with corticosteroid (7.4 vs. 19.6%, *P* = 0.037) were found between the I-BMD and NI-BMD groups (Table 1). The other baseline characteristics did not differ between the two groups. None of the variables had an absolute correlation coefficient greater than 0.7 (ESM1).

**Table 1:** Patient characteristics before PTx

	All patients	NI-BMD	I-BMD	*P* value
	(n = 173)	(n = 92)	(n = 81)	
Age (years)	58 (47–63)	58 (47–64)	57 (47–63)	.534
Sex (male/female)	81/92	42/50	39/42	.861
HD/PD	161/9	87/4	74/5	.827
Dialysis vintage (months)	117 (76–170)	116 (88–172)	120 (66–161)	.430
BMI (kg/m^2^)	21.5 (19.4–24.4)	21.8 (20.0–24.6)	20.9 (18.9–23.8)	.164
History of fracture (%)	5 (2.9)	3 (3.3)	2 (2.5)	1.000
Renal diagnosis (%)				.526
Glomerulonephritis	101 (58.4)	53 (57.6)	48 (59.3)	
Diabetic nephropathy	26 (15.0)	13 (14.1)	13 (16.0)	
Congenital/hereditary disease	11 (6.4)	7 ( 7.6)	4 ( 4.9)	
Hypertension	17 (9.8)	9 ( 9.8)	8 ( 9.9)	
Others	5 (2.9)	1 ( 1.1)	4 ( 4.9)	
Unknown	13 (7.5)	9 ( 9.8)	4 ( 4.9)	
Laboratory data				
Alb (g/dL)	4.3 (4.0–4.5)	4.3 (4.0–4.5)	4.2 (4.0–4.5)	.733
Corrected Ca (mg/dL)	10.2 (9.5–10.7)	10.4 (9.8–11.0)	9.9 (9.2–10.5)	**.002**
P (mg/dL)	5.0 (4.0–6.1)	4.7 (3.8–6.1)	5.1 (4.2–6.1)	.145
i-PTH (pg/mL)	476.5 (260.0–846.8)	456.0 (259.0–808.0)	547.0 (269.5–930.0)	.262
BAP (μg/L)	28.6 (18.8–43.6)	23.1 (17.2–36.5)	32.3 (23.7–54.9)	**.002**
1,25-OHD (pg/mL)	23.7 (11.4–38.7)	21.3 (13.0–39.3)	23.8 (11.4–38.4)	.693
BMD in the lumbar spine (g/cm^2^)	0.63 (0.56–0.74)	0.65 (0.57–0.75)	0.61 (0.52–0.72)	.077
T-score at the lumbar spine	− 2.7 (− 3.8 to − 1.2)	− 2.5 (− 3.3 to − 1.0)	− 3.0 (− 3.9 to − 1.6)	**.047**
BMD in the radius (g/cm^2^)	0.44 (0.38–0.52)	0.47 (0.37–0.52)	0.42 (0.38–0.49)	.101
T-score at the radius	− 3.5 (− 4.6 to − 2.5)	− 3.2 (− 4.3 to − 2.0)	− 3.7 (− 4.8 to − 2.9)	**.019**
Medical therapy				
Cinacalcet HCL (mg/day)				.243
0	73 (42.2)	44 (47.8)	29 (35.8)	
< 50	40 (23.1)	18 (19.6)	22 (27.2)	
≥ 50	60 (34.7)	30 (32.6)	30 (37.0)	
VDRA (%)	133 (76.9)	71 (77.2)	62 (76.5)	.922
Bisphosphonate (%)	3 (1.7)	1 (1.1)	2 (2.5)	.911
Corticosteroid (%)	24 (13.9)	18 (19.6)	6 (7.4)	**.037**

*P* compares the NI-BMD and I-BMD groups

Data for continuous variables are presented as median (interquartile range)

*1,25-OHD*, 1,25-dihydroxy vitamin D; *Alb*, albumin; *BAP*, bone-specific alkaline phosphatase; *BMD*, bone mineral density; *BMI*, body mass index; *Ca*, calcium; *Cinacalcet HCl*, cinacalcet hydrochloride; *HD*, hemodialysis; *I-BMD*, bone mineral density increased by ≥ 10% after parathyroidectomy; *i-PTH*, intact parathyroid hormone; *NI-BMD*, bone mineral density increased by < 10% after parathyroidectomy; *P*, phosphorus; *PD*, peritoneal dialysis; *PTx*, parathyroidectomy; *VDRA*, vitamin D receptor activator

### Postoperative outcomes

Overall, at 1-year post-PTx, the median BMD in the lumbar spine was increased by 8.7%. The I-BMD group achieved a significant improvement in lumbar T-score post-PTx (− 3.0 before PTx to − 1.4 in 1-year post-PTx). None of the patients received any additional treatment for osteoporosis within 1-year post-PTx but received vitamin D and Ca supplementation. The sCa levels on the first day post-PTx (8.4 vs. 8.9 mg/dL, *P* = 0.001) and the intact PTH 1-year post-PTx (21.0 vs. 48.0 pg/mL, *P* < 0.001) were significantly lower in the I-BMD group than in the NI-BMD group (Table 2). In the lumbar spine, post-PTx BMD (0.73 vs. 0.64 g/cm^2^, *P* < 0.001), T-score (− 1.4 vs. − 2.4, *P* < 0.001) and percent change of BMD (19.0 vs 0.7%, *P* < 0.001) were significantly higher in the I-BMD group than in the NI-BMD group (Table 2).

**Table 2:** Postoperative outcomes

	All patients	NI-BMD	I-BMD	*P*- value
	(n = 173)	(n = 92)	(n = 81)	
Number of parathyroid glands resected at PTx	4 (4–4)	4 (4–4)	4 (4–4)	.430
Total weight of the parathyroid glands (mg)	1396 (900–2079)	1444 (887–1828)	1321 (933–2196)	.781
Min-PTH (pg/mL)	5.0 (3.0–10.0)	5.0 (3.0–8.0)	5.0 (4.0–11.0)	.538
Ca on the first day post-PTx (mg/dL)	8.8 (8.2–9.2)	8.9 (8.5–9.3)	8.4 (9.0–8.9)	**.001**
Ca supplementation at discharge (mg/day)	1200 (600–2400)	1200 (600–1800)	1200 (600–2400)	.065
Alfacalcidol at discharge (μg/day)	1.00 (1.00–1.75)	1.00 (1.00–1.00)	1.00 (1.00–2.00)	.096
i-PTH at 1-year post-PTx (pg/mL)	29.5 (15.8–63.3)	48.0 (21.8–71.3)	21.0 (12.0–46.3)	** < .001**
Post-PTx BMD in the lumbar spine (g/cm^2^)	0.68 (0.61–0.81)	0.64 (0.58–0.73)	0.73 (0.66–0.90)	** < .001**
Post-PTx T-score at the lumbar spine	− 2.1 (− 3.0 to − 0.4)	− 2.4 (− 3.5 to − 1.5)	− 1.4 (− 2.5–1.0)	** < .001**
Percent change in BMD in the lumbar spine (%)	8.7 (− 0.2–18.0)	0.7 (− 5.5–5.9)	19.0 (14.4–26.8)	** < .001**
Post-PTx BMD in the radius (g/cm^2^)	0.45 (0.39–0.52)	0.47 (0.9–0.52)	0.43 (0.38–0.50)	.249
Post-PTx T-score at the radius	− 3.3 (− 4.3 to − 2.3)	− 3.1 (− 4.1 to − 2.0)	− 3.6 (− 4.6 to − 2.6)	.080
Percent change in BMD in the radius (%)	1.3 (− 0.7–3.7)	1.0 (− 1.5–3.0)	2.2 (0.5–4.4)	**.034**
Time from PTx to post-PTx BMD examination (months)	12 (11–13)	12 (11–13)	12 (11–13)	.586
Any fracture after PTx (%)	4 (2.3)	2 (2.2)	2 (2.5)	1.000
Follow-up after PTx (months)	40 (25–73)	38 (24–78)	46 (25–64)	.815

*P* compares the NI-BMD and I-BMD groups

Data for the continuous variables are presented as median (interquartile range)

*BMD*, bone mineral density; *I-BMD*, bone mineral density increased by 10% or more after parathyroidectomy; *i-PTH*, intact parathyroid hormone; *Min-PTH*, parathyroid hormone on the first day after parathyroidectomy; *NI-BMD*, bone mineral density increased by less than 10% after parathyroidectomy; *PTx*, parathyroidectomy

### Logistic regression analyses

**Fig. 2:**
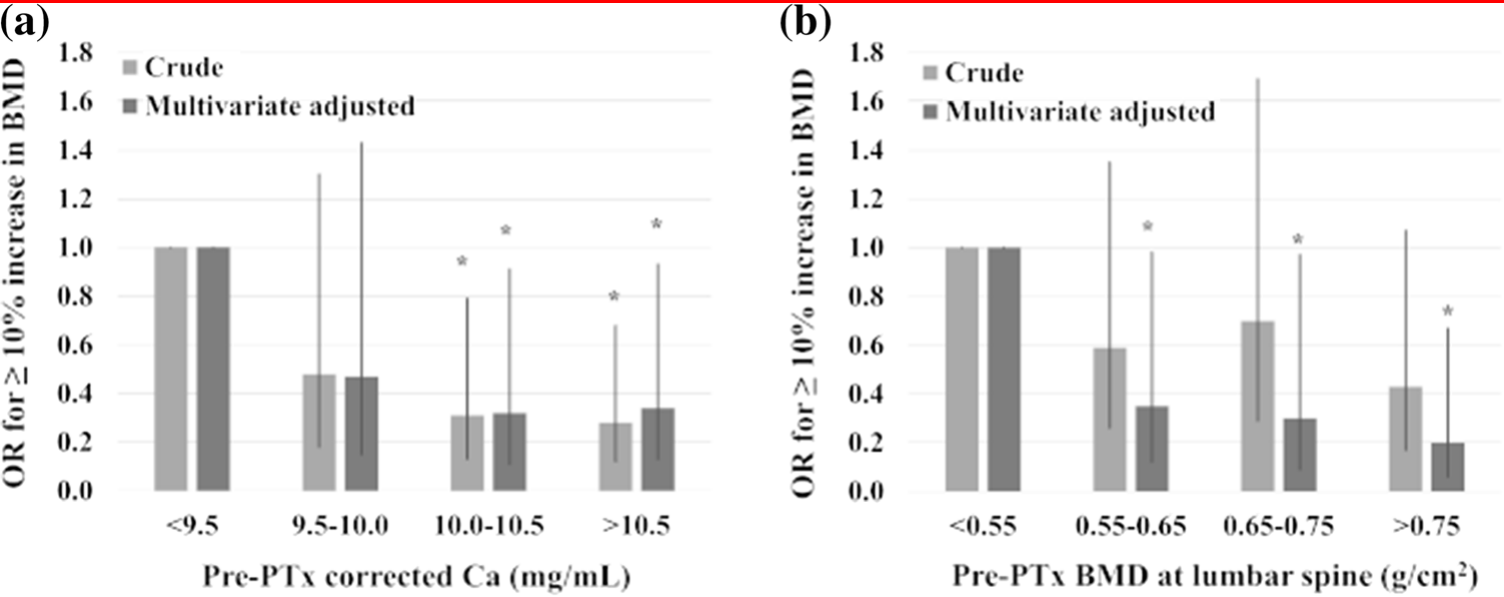
ORs and 95% CIs for ≥ 10% increase in BMD in the lumbar spine according to the categories of pre-PTx sCa levels and pre-PTx BMD in the lumbar spine using crude and multivariable-adjusted logistic models. **a** ORs according to the categories of pre-PTx sCa levels. The multivariable-adjusted analysis included age, BMI, Log BAP, pre-PTx BMD in the lumbar spine and treatment with corticosteroid. **b** ORs according to categories of pre-PTx BMD in the lumbar spine. The multivariable-adjusted analysis included age, BMI, Log BAP, pre-PTx sCa and treatment with corticosteroid. *P < 0.05. *BAP*, bone-specific alkaline phosphatase; *BMD*, bone mineral density; *BMI*, body mass index; *95% CI*, 95% confidence interval; *Log*, logarithm; *OR*, odds ratio; *PTx*, parathyroidectomy; *sCa*, serum calcium

The univariate logistic regression analysis demonstrated that pre-PTx sCa (*P* = 0.003; OR, 0.572; 95% confidence interval [CI]: 0.397–0.824), Log pre-PTx BAP (*P* = 0.013; OR, 1.695; 95% CI: 1.117–2.572) and treatment with corticosteroid (*P* = 0.026; OR, 0.329; 95% CI: 0.124–0.875) had significant associations with ≥ 10% increase in BMD post-PTx. The multivariate logistic regression analysis revealed that age ≥ 70 years (*P* = 0.005; OR, 0.138; 95% CI: 0.034–0.555), pre-PTx sCa (*P* = 0.017; OR, 0.598; 95% CI: 0.392–0.911), pre-PTx BMD in the lumbar spine (*P* = 0.003; OR, 0.013; 95% CI: 0.001–0.229) and corticosteroid use (*P* = 0.002; OR, 0.153; 95% CI: 0.045–0.514) were negatively associated with ≥ 10% increase in BMD post-PTx (Table 3). Figure 2 shows the unadjusted and multivariable-adjusted ORs for ≥ 10% increase in BMD post-PTx associated with the categories of pre-PTx sCa and BMD values. The multivariate adjusted OR of ≥ 10% increase in BMD significantly decreased when the sCa levels were within the range of 10.0–10.4 mg/dL (OR, 0.319; 95% CI: 0.112–0.912) and ≥ 10.5 mg/dL (OR, 0.343; 95% CI: 0.127–0.927; Fig. 2). Similarly, a downhill relationship was observed with a significant decrease in the multivariate adjusted OR of ≥ 10% increase in BMD, with pre-PTx BMD values of 0.55–0.64 g/cm^2^ (OR, 0.349; 95% CI: 0.124–0.979), 0.65–0.74 g/cm^2^ (OR, 0.302; 95% CI: 0.094–0.970) and ≥ 0.75 g/cm^2^ (OR, 0.203; 95% CI: 0.061–0.670; Fig. 2).

**Table 3:** Logistic regression for ≥ 10% increases in BMD in the lumbar spine post-PTx

Factors	Univariate			Multivariate		
	OR	95% CI	*P* value	OR	95% CI	*P* value
Age (years, reference to < 50 years)						
50–59	1.364	0.629 –2.955	.432	0.812	0.328–2.011	.652
60–69	1.013	0.461–2.226	.974	0.457	0.172–1.215	.117
≥ 70	0.492	0.150–1.615	.242	0.138	0.034–0.555	**.005**
Male sex	1.105	0.608–2.011	.743			
Peritoneal dialysis	1.470	0.381–5.674	.576			
Dialysis vintage (months)	0.999	0.995–1.003	.485			
BMI (kg/m^2^)	0.951	0.871–1.037	.255	0.907	0.814–1.011	.077
Diabetes	1.162	0.504–2.676	.725			
History of fracture	0.751	0.122–4.610	.757			
Pre-PTx Alb (g/dL)	0.910	0.405–2.047	.820			
Pre-PTx corrected Ca (mg/dL)	0.572	0.397–0.824	**.003**	0.598	0.392–0.911	**.017**
Pre-PTx P (mg/dL)	1.161	0.967–1.394	.110			
Log pre-PTx i-PTH (pg/mL)	1.266	0.814–1.970	.295			
Log pre-PTx BAP (μg/L)	1.695	1.117–2.572	**.013**	1.570	0.967–2.549	.068
Pre-PTx BMD in the lumbar spine (g/cm^2^)	0.162	0.021–1.257	.082	0.013	0.001–0.229	**.003**
Cinacalcet HCL (mg/day, reference to 0 mg/day)						
< 50	1.854	0.850–4.044	.121			
≥ 50	1.517	0.761–3.024	.236			
Vitamin D receptor activator	0.965	0.475–1.959	.922			
Bisphosphonate	2.304	0.205–25.890	.499			
Corticosteroid	0.329	0.124–0.875	**.026**	0.153	0.045–0.514	**.002**

*Alb*, albumin; *BAP*, bone-specific alkaline phosphatase; *BMD*, bone mineral density; *BMI*, body mass index; *Ca*, Calcium; *95% CI*, 95% confidence interval; *Cinacalcet HCl*, cinacalcet hydrochloride; *i-PTH*, intact parathyroid hormone; *Log*, logarithm; *OR*, odds ratio; *P*, phosphorus; *PTx*, parathyroidectomy

### Discussion

Increase in BMD post-PTx probably contributes to the reduction in fracture risk among patients with advanced SHPT [9, 10, 21, 22]. We retrospectively examined 173 SHPT patients to clarify the predictive factors for post-PTx BMD improvement. In the present study, a multivariate logistic regression analysis revealed that age, sCa level and pre-PTx BMD are possible predictors of BMD improvement post-PTx in SHPT patients. The multivariate analysis also revealed that corticosteroid treatment was negatively associated with post-PTx BMD changes. The results obtained from the logistic regression analyses were also supported by those of the multivariate linear regression analysis of post-PTx BMD-to-pre-PTx BMD ratio in the lumbar spine (ESM 2). Some previous studies also reported BMD changes after PTx for SHPT. In a study with 15 SHPT patients, Yano et al. reported PTH and ALP levels as predictors of post-PTx BMD changes [11]. Another study with 26 SHPT patients by Lu et al. showed that BMD changes correlated negatively with BAP and correlated positively with tartrate-resistant acid phosphatase type 5b [12]. Moreover, Li et al. conducted a study in 34 SHPT patients, which demonstrated that parathyroid weight and pre-PTx BMD could be used to predict BMD changes [13]. However, the sample sizes in these studies were too small for a multivariate analysis. Conversely, several studies with relatively large sample sizes have focused on post-PTx BMD changes in patients with primary hyperparathyroidism (PHPT) [17, 23]. Sharma et al. demonstrated that age, sex and pre-PTx BMD had a significant association with BMD changes post-PTx in 123 PHPT patients [17]. Furthermore, a correlation between pre-PTx sCa and post-PTx BMD changes was reported by Lee et al. in a study with 92 PHPT patients [23].

In the present study, elderly patients exhibited a poor improvement in post-PTx BMD, which is consistent with the report by Sharma et al. [17]. Since physical activity has been linked to increased bone mass in osteoporotic patients [24], it seems likely to hypothesize that the decline in physical activity with age might have hindered BMD improvement in our elderly subjects. In addition, aging itself reportedly decreased bone turnover [25].

To the best of our knowledge, the significant association observed herein between lower pre-PTx sCa levels and post-PTx BMD improvement in SHPT patients is a novel finding. In the present study, the lower sCa levels in the I-BMD group may reflect the extent of Ca adsorption onto bones [26]. In addition, lower pre-PTx sCa level probably increases the frequency of post-PTx hypocalcemia and doses of Ca and vitamin D supplementation, which may in turn contribute to enhanced bone mass. Furthermore, supplementation with a higher dose of vitamin D post-PTx might contribute to not only the extent in BMD change but also the difference in post-PTx PTH levels between the I-BMD and NI-BMD groups because of the known suppressive effect of vitamin D on PTH [27].

The association we established between lower pre-PTx BMD values and greater post-PTx BMD increase rates concurs with some previous reports [7, 14, 28]. The results of these studies serve as strong evidence supporting PTx in SHPT patients with bone loss. Although calcimimetics are effective in improving BMD, a past randomized study has shown PTx to be superior to calcimimetics on this front [29]. Therefore, PTx could be considered for SHPT patients with osteoporosis, even those with well-controlled biochemical parameters.

The major limitations of the present study were its single-center retrospective nature and potential selection bias arising from the study design. Furthermore, data on bone metabolism markers other than BAP were not obtained. In addition, there was a lack in long-term data on Ca and vitamin D supplementation and patients’ daily activities after surgery. Further studies with larger sample sizes and longer observation periods are expected to validate the findings of our study.

In conclusion, age (≥ 70 years) could negatively impact post-PTx bone density improvements whereas presurgical lower Ca levels and bone mineral density could predict enhancements in bone mass a year after PTx. Earlier surgical intervention should be considered for patients with advanced hyperparathyroidism and marked osteopenia and is expected to achieve positive outcomes in terms of bone mass in patients without hypercalcemia.

## Supplementary Information

Below is the link to the electronic supplementary material.

Supplementary file1 (PDF 204 kb)

Supplementary file2 (PDF 175 kb)
