# Supplementary material for: Predictors of Bone Mineral Density Improvement after Parathyroidectomy for Secondary Hyperparathyroidism: A Retrospective Single-Center Analysis
Source: World J Surg. 2021 Jun 16;45(9):2777–84. doi: 10.1007/s00268-021-06186-1 (PMC8322001; doi:10.1007/s00268-021-06186-1)
Supplement: Supplementary file 1 — Supplementary file1 (PDF 204 kb) [file 268_2021_6186_MOESM1_ESM.pdf]

Predictors of bone mineral density improvement after parathyroidectomy for secondary hyperparathyroidism: a retrospective single-center analysis

World journal of Surgery

Manabu Okada, M.D., Ph.D.1, Yoshihiro Tominaga, M.D., Ph.D.1, Toshihide Tomosugi, M.D.1, Takahisa Hiramitsu, M.D., Ph.D.1, Toshihiro Ichimori, M.D.1, Tetsuhiko Sato, M.D., Ph.D.

Corresponding author: Manabu Okada, M.D., Ph.D.
Department of Transplantation and Endocrine Surgery,
Nagoya Daini Red Cross Hospital, 2-9 Myoken-cho,
Showa-ku, Nagoya, Japan 4668650
Tel: 81-528321121 Fax: 81-528321130 E-mail: ubanam@nagoya2.jrc.or.jp

EMS 1 Spearman rank correlation coefficient

		Age 0：-49 1：50-59 2：60−69 3：70−	0:Female1:Male	Dialysis duration	BMI	VDRA	I-PTH	Alb	sCa	sP	BMD at Lumbar Spine	BAP	DM	Cincacalcet 0 1:25mg 2:50mg 3:>50mg	Bisphosphonate	Corticosteroid	0:HD1:PD
Age 0：-49 1：50-59 2：60−69 3：70−	Correlation coefficient	1.000	-0.040	.202**	-0.100	.180*	0.087	-.417**	0.043	-0.010	-.344**	-0.029	-0.039	-.197**	-0.077	-.176*	-0.137
	P value		0.599	0.008	0.189	0.018	0.264	0.000	0.575	0.893	0.000	0.703	0.613	0.010	0.312	0.020	0.074
	Number	173	173	173	173	173	168	169	169	169	173	173	173	173	173	173	170
0:Female1:Male	Correlation coefficient	-0.040	1.000	0.006	.153*	0.130	0.016	.168*	-0.034	0.066	.260**	-.150*	0.059	0.006	0.053	-0.108	-0.115
	P value	0.599		0.934	0.045	0.088	0.841	0.029	0.662	0.395	0.001	0.050	0.439	0.935	0.490	0.155	0.136
	Number	173	173	173	173	173	168	169	169	169	173	173	173	173	173	173	170
Dialysis duration	Correlation coefficient	.202**	0.006	1.000	-.279**	.253**	-.205**	0.024	.192*	0.042	-0.087	-.155*	-.188*	-0.098	0.004	-0.117	-.179*
	P value	0.008	0.934		0.000	0.001	0.008	0.757	0.013	0.590	0.257	0.042	0.013	0.197	0.963	0.127	0.020
	Number	173	173	173	173	173	168	169	169	169	173	173	173	173	173	173	170
BMI	Correlation coefficient	-0.100	.153*	-.279**	1.000	0.113	.155*	-0.111	-.214**	0.070	.198**	-0.078	.348**	0.104	-0.050	-.150*	0.090
	P value	0.189	0.045	0.000		0.137	0.045	0.151	0.005	0.365	0.009	0.310	0.000	0.175	0.516	0.048	0.242
	Number	173	173	173	173	173	168	169	169	169	173	173	173	173	173	173	170
VDRA	Correlation coefficient	.180*	0.130	.253**	0.113	1.000	-0.033	0.007	-0.076	.247**	0.146	-.314**	0.116	.191*	-0.137	-.613**	-.188*
	P value	0.018	0.088	0.001	0.137		0.672	0.924	0.326	0.001	0.055	0.000	0.130	0.012	0.072	0.000	0.014
	Number	173	173	173	173	173	168	169	169	169	173	173	173	173	173	173	170
I-PTH	Correlation coefficient	0.087	0.016	-.205**	.155*	-0.033	1.000	-0.093	-.302**	.280**	-0.014	.379**	-0.001	-0.075	-0.090	-.212**	-0.042
	P value	0.264	0.841	0.008	0.045	0.672		0.230	0.000	0.000	0.860	0.000	0.988	0.335	0.244	0.006	0.590
	Number	168	168	168	168	168	168	168	168	168	168	168	168	168	168	168	165
Alb	Correlation coefficient	-.417**	.168*	0.024	-0.111	0.007	-0.093	1.000	.153*	-0.077	.236**	0.021	-0.061	0.085	-0.020	0.085	-0.118
	P value	0.000	0.029	0.757	0.151	0.924	0.230		0.047	0.321	0.002	0.791	0.428	0.274	0.798	0.271	0.129
	Number	169	169	169	169	169	168	169	169	169	169	169	169	169	169	169	166
sCa	Correlation coefficient	0.043	-0.034	.192*	-.214**	-0.076	-.302**	.153*	1.000	-.237**	-0.030	-0.109	-0.099	-.256**	-0.034	.210**	0.004
	P value	0.575	0.662	0.013	0.005	0.326	0.000	0.047		0.002	0.700	0.158	0.202	0.001	0.656	0.006	0.960
	Number	169	169	169	169	169	168	169	169	169	169	169	169	169	169	169	166
sP	Correlation coefficient	-0.010	0.066	0.042	0.070	.247**	.280**	-0.077	-.237**	1.000	.193*	-.161*	0.031	0.140	-0.061	-.503**	-.156*
	P value	0.893	0.395	0.590	0.365	0.001	0.000	0.321	0.002		0.012	0.036	0.691	0.069	0.433	0.000	0.044
	Number	169	169	169	169	169	168	169	169	169	169	169	169	169	169	169	166
BMD at Lumbar Spine	Correlation coefficient	-.344**	.260**	-0.087	.198**	0.146	-0.014	.236**	-0.030	.193*	1.000	-.168*	0.024	.162*	-0.095	-.251**	-0.023
	P value	0.000	0.001	0.257	0.009	0.055	0.860	0.002	0.700	0.012		0.027	0.756	0.033	0.214	0.001	0.766
	Number	173	173	173	173	173	168	169	169	169	173	173	173	173	173	173	170
BAP	Correlation coefficient	-0.029	-.150*	-.155*	-0.078	-.314**	.379**	0.021	-0.109	-.161*	-.168*	1.000	-0.115	-0.054	-0.082	.218**	0.133
	P value	0.703	0.050	0.042	0.310	0.000	0.000	0.791	0.158	0.036	0.027		0.133	0.483	0.286	0.004	0.084
	Number	173	173	173	173	173	168	169	169	169	173	173	173	173	173	173	170
DM	Correlation coefficient	-0.039	0.059	-.188*	.348**	0.116	-0.001	-0.061	-0.099	0.031	0.024	-0.115	1.000	-0.107	-0.056	-0.028	-0.024
	P value	0.613	0.439	0.013	0.000	0.130	0.988	0.428	0.202	0.691	0.756	0.133		0.162	0.465	0.711	0.756
	Number	173	173	173	173	173	168	169	169	169	173	173	173	173	173	173	170
Cincacalcet 0 1:25mg 2:50mg 3:>50mg	Correlation coefficient	-.197**	0.006	-0.098	0.104	.191*	-0.075	0.085	-.256**	0.140	.162*	-0.054	-0.107	1.000	-0.087	-.238**	-0.023
	P value	0.010	0.935	0.197	0.175	0.012	0.335	0.274	0.001	0.069	0.033	0.483	0.162		0.253	0.002	0.767
	Number	173	173	173	173	173	168	169	169	169	173	173	173	173	173	173	170
Bisphosphonate	Correlation coefficient	-0.077	0.053	0.004	-0.050	-0.137	-0.090	-0.020	-0.034	-0.061	-0.095	-0.082	-0.056	-0.087	1.000	.203**	-0.032
	P value	0.312	0.490	0.963	0.516	0.072	0.244	0.798	0.656	0.433	0.214	0.286	0.465	0.253		0.007	0.682
	Number	173	173	173	173	173	168	169	169	169	173	173	173	173	173	173	170
Corticosteroid	Correlation coefficient	-.176*	-0.108	-0.117	-.150*	-.613**	-.212**	0.085	.210**	-.503**	-.251**	.218**	-0.028	-.238**	.203**	1.000	.214**
	P value	0.020	0.155	0.127	0.048	0.000	0.006	0.271	0.006	0.000	0.001	0.004	0.711	0.002	0.007		0.005
	Number	173	173	173	173	173	168	169	169	169	173	173	173	173	173	173	170
0:HD1:PD	Correlation coefficient	-0.137	-0.115	-.179*	0.090	-.188*	-0.042	-0.118	0.004	-.156*	-0.023	0.133	-0.024	-0.023	-0.032	.214**	1.000
	P value	0.074	0.136	0.020	0.242	0.014	0.590	0.129	0.960	0.044	0.766	0.084	0.756	0.767	0.682	0.005	
	Number	170	170	170	170	170	165	166	166	166	170	170	170	170	170	170	170
