# Supplementary material for: Predictors of Bone Mineral Density Improvement after Parathyroidectomy for Secondary Hyperparathyroidism: A Retrospective Single-Center Analysis
Source: World J Surg. 2021 Jun 16;45(9):2777–84. doi: 10.1007/s00268-021-06186-1 (PMC8322001; doi:10.1007/s00268-021-06186-1)
Supplement: Supplementary file 2 — Supplementary file2 (PDF 175 kb) [file 268_2021_6186_MOESM2_ESM.pdf]

Predictors of bone mineral density improvement after parathyroidectomy for secondary hyperparathyroidism: a retrospective single-center analysis

World journal of Surgery

Manabu Okada, M.D., Ph.D.[1], Yoshihiro Tominaga, M.D., Ph.D.[1], Toshihide Tomosugi, M.D.[1], Takahisa Hiramitsu, M.D., Ph.D.[1], Toshihiro Ichimori, M.D.[1], Tetsuhiko Sato, M.D., Ph.D.

Corresponding author: Manabu Okada, M.D., Ph.D.

Department of Transplantation and Endocrine Surgery,

Nagoya Daini Red Cross Hospital, 2-9 Myoken-cho,

Showa-ku, Nagoya, Japan 4668650

Tel: 81-528321121   Fax: 81-528321130   E-mail: ubanam@nagoya2.jrc.or.jp

ESM 2 Linear regression analysis for log post-PTx BMD-to-pre-PTx BMD ratio in the lumbar spine

	**Univariate**				**Multivariate**			R2 = 0.34
Log post-PTx BMD-to-pre-PTx BMD ratio	ERC (95% CI)	SE	*T* value	*P* value	ERC (95% CI)	SE	*T* value	*P* value
Age (years, reference to < 50 years)								
50–59	0.01 (-0.04 – 0.07)	0.02	0.46	.647	-0.02 (-0.07 – 0.03)	0.03	-0.77	.444
60–69	0.00 (-0.05 – 0.06)	0.03	0.15	.880	-0.05 (-0.11 – 0.01)	0.03	-1.70	.091
≥ 70	0.00 (-0.08 – 0.08)	0.04	0.68	.965	-0.09 (-0.18 – -0.01)	0.04	-2.18	**.031**
Male sex	0.00 (-0.04 – 0.05)	0.02	0.21	.834	0.03 (0.00 – 0.07)	0.02	1.73	.085
Peritoneal dialysis	0.04 (-0.06 – 0.13)	0.05	0.78	.436	0.04 (-0.06 – 0.13)	0.05	0.80	.426
Dialysis vintage (months)	0.00 (0.00 – 0.00)	0	-0.98	.329	0.00 (0.00 – 0.00)	0.00	-0.41	.681
BMI (kg/m^2)	0.00 (-0.01 – 0.00)	0	-0.59	.559	0.00 (-0.01 – 0.00)	0.00	-0.84	.404
Diabetes	0.02 (-0.04 – 0.08)	0.03	0.75	.454	0.02 (-0.04 – 0.08)	0.03	0.53	.624
Pre-PTx Alb (g/dL)	0.02 (-0.08 – 0.03)	0.03	-0.81	.416	0.00 (-0.06 – 0.06)	0.03	-0.09	.930
Pre-PTx Ca (mg/dL)	-0.04 (-0.06 – -0.02)	0.01	-3.35	**<.001**	-0.04 (-0.06 – -0.01)	0.01	-2.98	**.003**
Pre-PTx P (mg/dL)	0.00 (-0.01 – 0.01)	0.01	-0.23	.822	0.00 (-0.01 – 0.01)	0.01	-0.05	.957
Log pre-PTx i-PTH (pg/mL)	0.01 (-0.02 – 0.04)	0.02	0.57	.566	-0.03 (-0.07 – 0.00)	0.02	-1.76	.080
Log pre-PTx BAP (μg/L)	0.06 (0.03 – 0.09)	0.02	3.91	**<.001**	0.06 (0.02 – 0.09)	0.02	3.26	**.001**
Pre-PTx BMD at lumbar spine(g/cm^2)	-0.34 (-0.50 – -0.17)	0.08	-4.10	**<.001**	-0.42 (-0.57 – -0.27)	0.08	-5.60	**<.001**
Cinacalcet HCL (mg/day, reference to 0 mg/day)								
<50	0.03 (-0.05 – 0.10)	0.04	0.67	.504	-0.01 (-0.07 – 0.04)	0.03	-0.54	.591
≥50	0.06 (−0.01 – 0.12)	0.03	1.64	.103	-0.03 (-0.08 – 0.02)	0.03	-1.03	.304
Vitamin D receptor activator	-0.01 (-0.06 – 0.04)	0.02	-0.56	.578	-0.02 (-0.08 – 0.04)	0.03	-0.78	.439
Bisphosphonate	0.09 (-0.07 – 0.24)	0.08	1.08	.280	0.07 (-0.07 – 0.22)	0.07	0.98	.327
Corticosteroid	-0.01 (-0.07 – 0.05)	0.03	-0.48	.635	-0.12 (-0.20 – -0.03)	0.04	-2.79	**.006**

The ratio of post-PTx BMD to pre-PTx BMD (post-/pre-PTx BMD ratio) was calculated as follows:
Post-/pre-PTx BMD ratio = post-PTx BMC (g/cm^2)/pre-PTx BMD (g/cm^2)
In the multivariate linear regression, 4 outliers from Log BAP and 5 outliers from Log post-/pre-PTx BMD ratio at lumbar spine were excluded using the Smirnov-Grubbs test to get the data closer to a normal distribution.
Alb, albumin; *BAP*, bone-specific alkaline phosphatase; *BMD*, bone mineral density; *BMI*, body mass index; *Ca*, Calcium; *95% CI*, 95% confidence interval; *Cinacalcet HCl*, cinacalcet hydrochloride; *ERC*, Estimated regression coefficient; *i-PTH*, intact parathyroid hormone; *Log*, logarithm; *P*, phosphorus; *PTx*, parathyroidectomy
